# Supplementary material for: The H163A mutation unravels an oxidized conformation of the SARS-CoV-2 main protease
Source: Nat Commun. 2023 Sep 12;14:5625. doi: 10.1038/s41467-023-40023-4 (PMC10497556; doi:10.1038/s41467-023-40023-4)
Supplement: Supplementary file 1 — Supplementary Information [file 41467_2023_40023_MOESM1_ESM.pdf]

The H163A Mutation Unravels an Oxidized Conformation of the SARS-CoV-2 Main Protease

SUPPLEMENTARY INFORMATION

Norman Tran[1†], Sathish Dasari[2†], Sarah Barwell[1], Matthew J. McLeod[3], Subha Kalyaanamoorthy[2], Todd Holyoak[1*], & Aravindhan Ganesan[4*]

[1]Department of Biology, Faculty of Science, University of Waterloo, 200 University Avenue West, Ontario N2L 3G1, Canada

[2]Department of Chemistry, Faculty of Science, University of Waterloo, 200 University Avenue West, Ontario N2L 3G1, Canada

[3]Physics Department, Cornell University, Ithaca, NY 14853, USA

[4]ArGan's Lab, School of Pharmacy, Faculty of Science, University of Waterloo, 10A Victoria Street South, Kitchener, Ontario N2G 1C5, Canada

[*]corresponding authors

Aravindhan Ganesan, aravindhan.ganesan@uwaterloo.ca
Todd Holyoak, tholyoak@uwaterloo.ca

[†]co-first authors

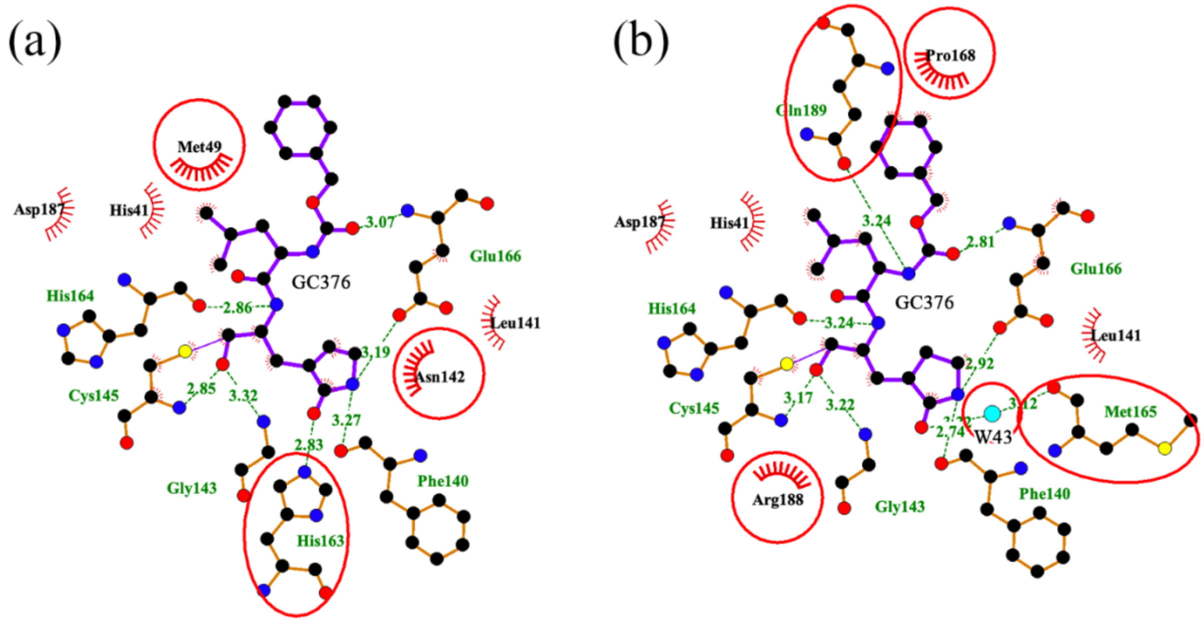

Supplementary Figure 1 – 2D Map of GC376-Mpro Interactions. GC376 generally interacts with the same residues for both the WT (a) and H163A mutant (b) enzymes. Many of these interactions are within the active-site pocket as GC376 is a covalent inhibitor. GC376 also makes a hydrogen bond with H163 in the lateral pocket. This interaction is substituted with W43 in the H163A structure. Circled residues highlight differences between the two structures. Hydrogen-bond distances are denoted in angstroms. This figure was made with LigPlot+ (version 2.2).[1]

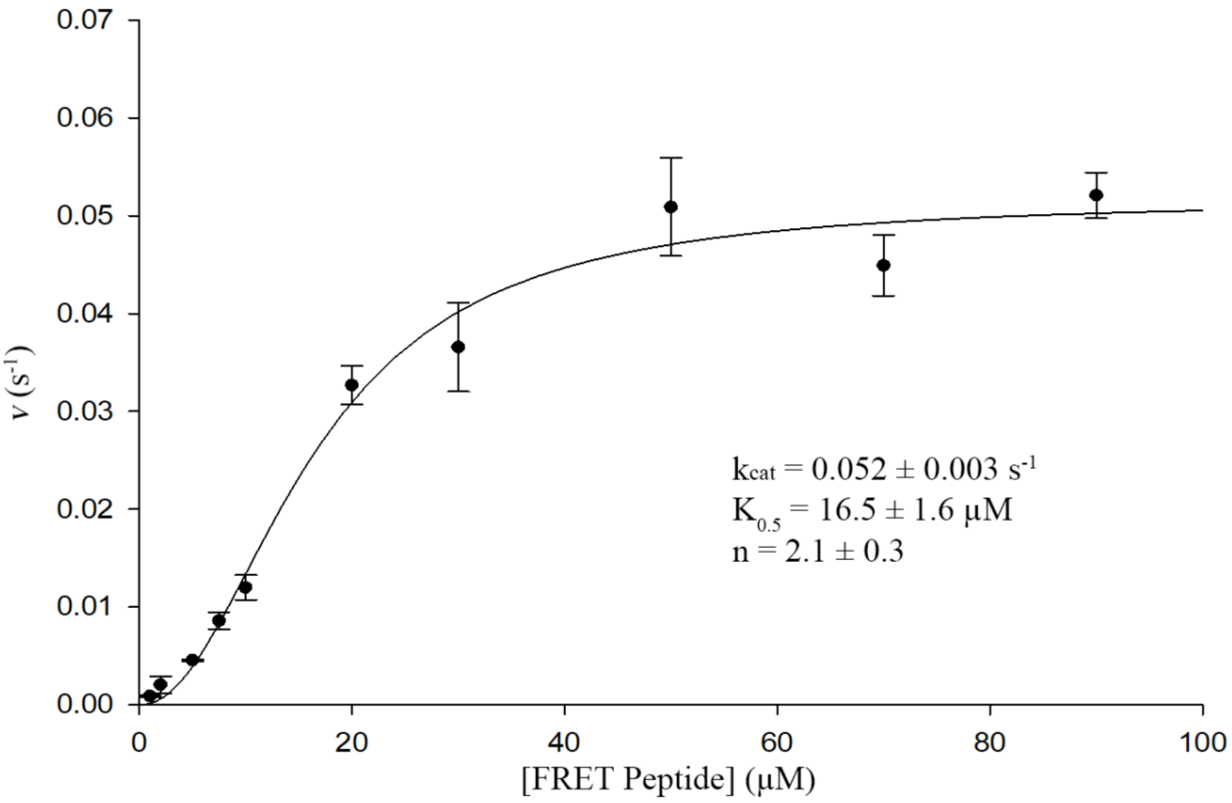

Supplementary Figure 2 – Michaelis-Menten Curve for Wild Type Mpro. Initial steady-state rates for WT Mpro were measured via a fluorescent-based kinetic assay at various concentrations of fluorescent peptide (n = 3 technically replicated samples; one of the replicates for 5 and 30 μM FRET peptide was removed). These data fit well to the Hill equation and showed positive kinetic cooperativity between the active sites of each monomer. Data are presented as mean values ± standard error of the mean. Source data are provided as a Source Data file.

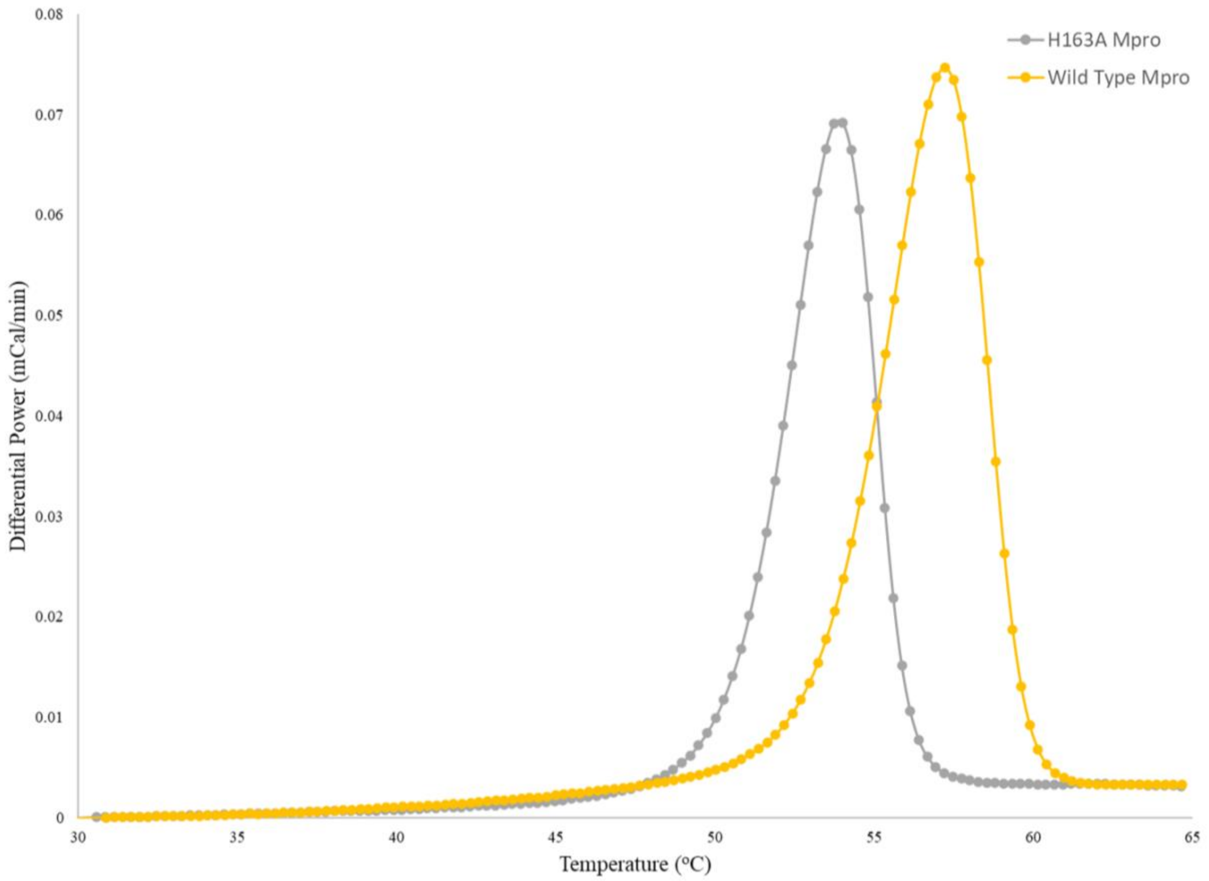

Supplementary Figure 3 – Differential Scanning Calorimetry Thermograms of Wild Type and H163A Mpro. The thermograms of WT and H163A Mpro show differences in thermal stability. Despite the peak shape fitting well to a concerted and oligomeric unfolding model, thermodynamic and mechanistic information about the enzymes' unfolding cannot be extracted from these data. Source data are provided as a Source Data file.

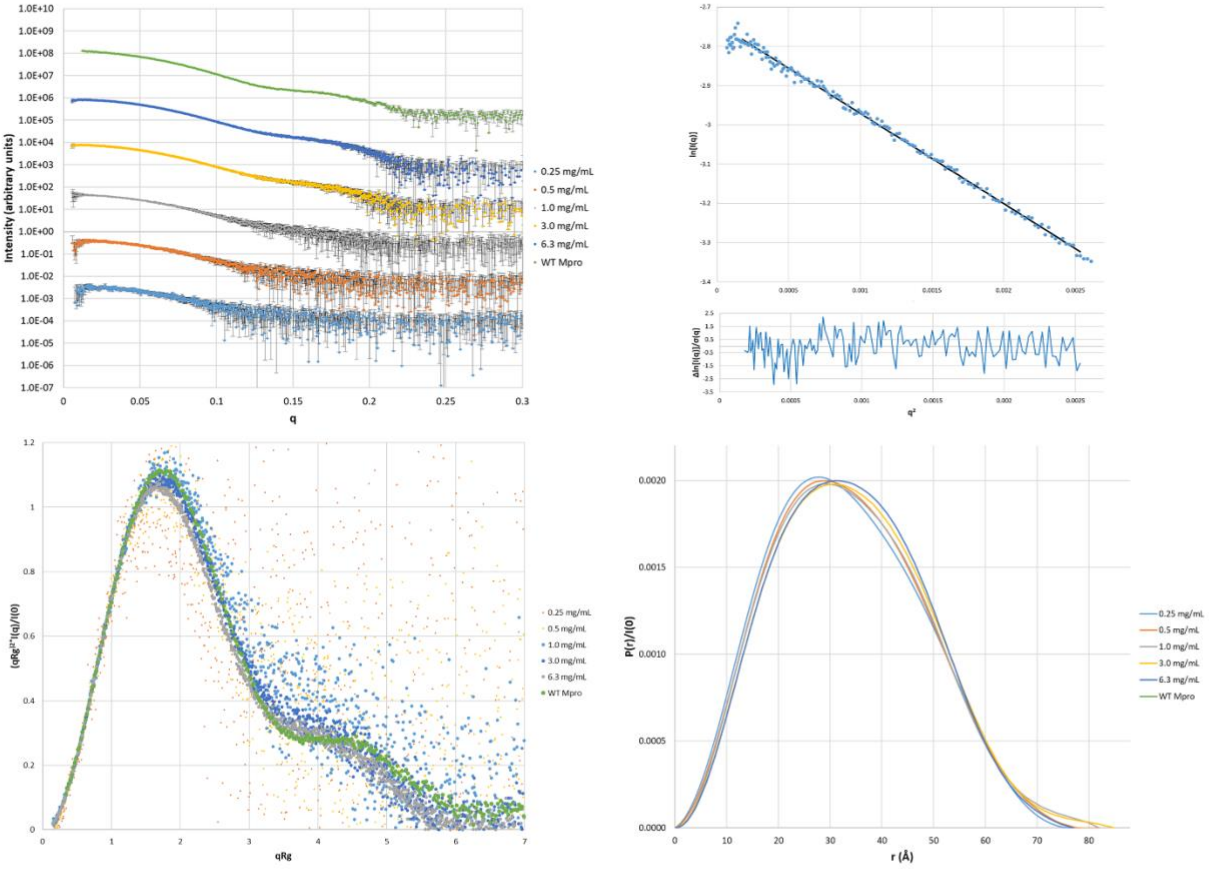

Supplementary Figure 4 – Comparing Small-Angle X-ray Scattering Profiles Between Wild Type and H163A Mpro. The scattering profiles of the H163A mutant are consistent in all regards to previously published dimeric WT Mpro SAXS data (SASBDB Entry SASDJG5).[2,3] The raw scattering curves (top left), normalized Kratky (bottom left), and normalized P(r) plots (bottom right) for various concentrations of H163A Mpro have the same general shape to WT Mpro, showing that the mutant scattering is consistent with a dimer for these concentrations. The good data quality of the representative 3.0 mg/mL data are exemplified by the linear Guinier region (top of top right plot) and the fit's non-skewed residuals (bottom of top right plot), which was fit to a $q_{max}R_g$ of ~1.32. Data were processed with RAW[4] and GNOM.[5] Raw scattering curves were offset in I(q) by factors of 50 to aid with visual comparison. Data in the raw scattering curves are presented as mean values ± one standard deviation across n=15 azimuthally averaged detector images. These errors were calculated by RAW,[4] which follows standard statistical best practices. Data points for the 0.25 and 0.5 mg/mL normalized P(r) plots are depicted as smaller points to reduce visual clutter due to the noisiness of the data. Source data are provided as a Source Data file.

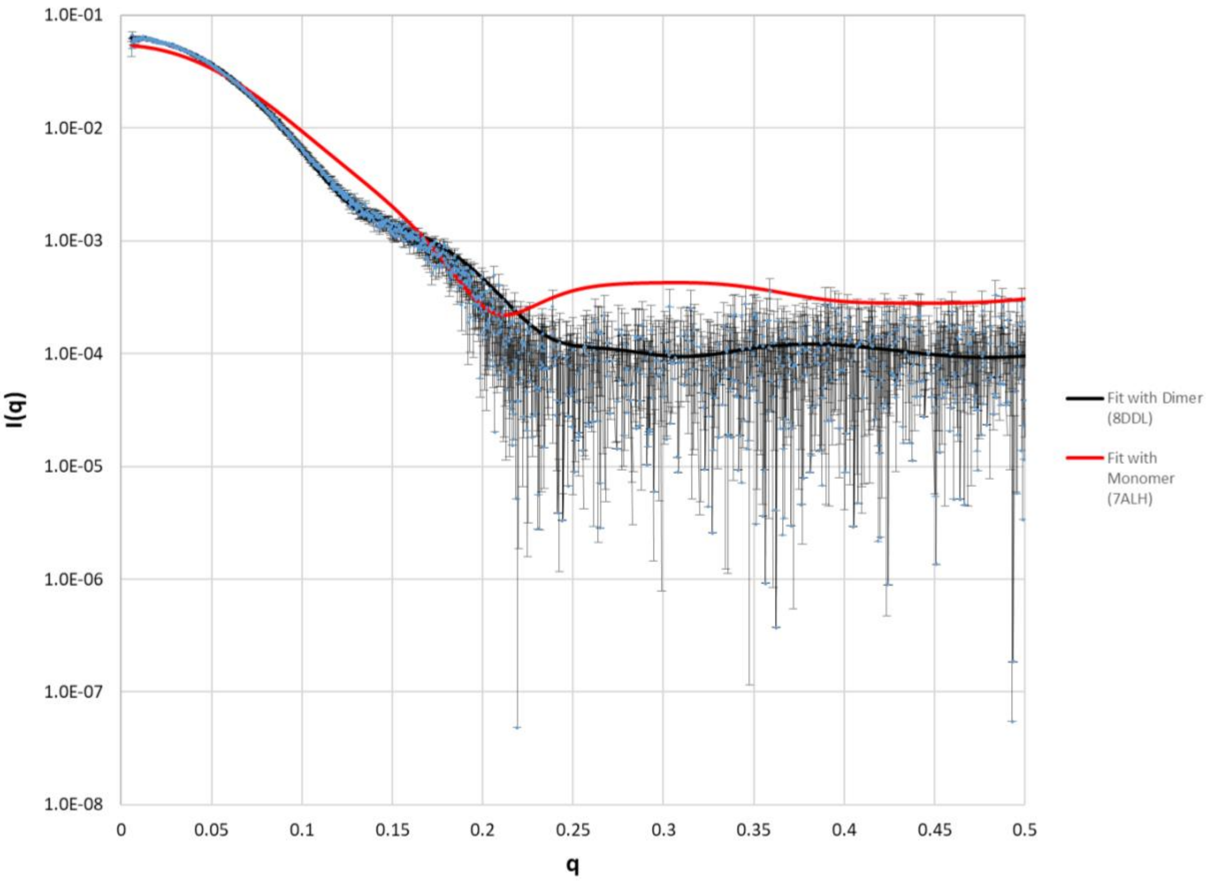

Supplementary Figure 5 – Fitting Simulated Scatter of Crystal Structures to H163A Mpro SAXS Data. Simulated scattering profiles generated from crystal structures of the H163A mutant dimer (black fit; PDB 8DDL; the dimer is the asymmetric unit) and a WT monomer model (red fit; PDB 7ALH; one protomer of the dimer is in the asymmetric unit) were fit to the experimental scattering profile of 3.0 mg/mL H163A Mpro using the FoXS webserver (accessed April 17th, 2023).[6] Data in the raw scattering curves are presented as mean values ± one standard deviation across n=15 azimuthally averaged detector images. These errors were calculated by RAW,[4] which follows standard statistical best practices. The simulated scatter from the H163A Mpro dimer reasonably fits the experimental data (χ^2 = 2.1) while the simulated scatter from the monomer model does not fit the data (χ^2 = 56.6). Source data are provided as a Source Data file.

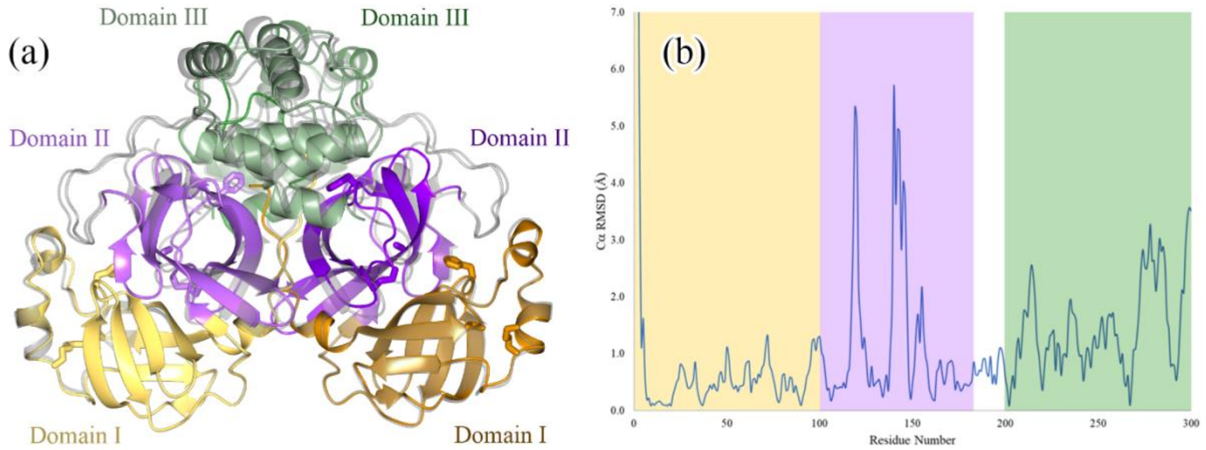

Supplementary Figure 6 – Global Comparisons Between Wild Type and H163A Mpro Structures. (a) Global differences are seen when comparing the structures of the WT (grey) and H163A mutant. This is most noticeable globally in Domain III (pale green/forest green), where several of the helices in the H163A structure are displaced relative to their positions in Domains I and II of WT Mpro. All molecular images were generated in CCP4MG.[7] (b) A comparison of Cα root-mean-squared deviation (RMSD) values between the WT and mutant residues. Cα RMSD values were calculated using Chimera (version 1.16-42360).[8] Due to the structural asymmetry between the two molecules in the asymmetric unit, Cα RMSD values were only calculated for chain B as it showed more structural deviation compared to chain A. (b) is colored with the same color scheme as (a). The most notable spikes in Cα RMSD for each domain, from the N- to C-terminus, correspond to a repositioning of the N-terminus in Domain I, rearrangement of the active-site and surrounding loops in Domain II, and an overall displacement of helices in Domain III. Source data are provided as a Source Data file.

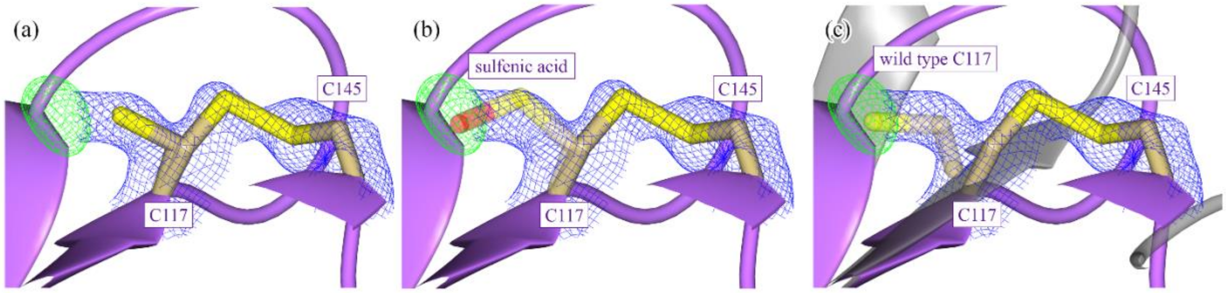

Supplementary Figure 7 – Accounting for the Positive Difference Density at C117 in the H163A Mutant Structure. (a) There is strong positive F_o-F_c density adjacent to the sulfur atom of C117 when the disulfide bond between C117 and C145 is broken. It is unclear whether this positive density is due to (b) an oxidized C117 as sulfenic acid or (c) a minor proportion of the beta strand containing C117 relaxing into its WT conformation (grey; PDB 7BB2), placing the WT C117 into the positive density. Because of this ambiguity and lack of direct evidence for either possibility, C117 is modelled as a reduced cysteine in the H163A mutant structure. Maps were rendered at 4.0 σ F_o-F_c and 1.2 σ $2F_o$-F_c.

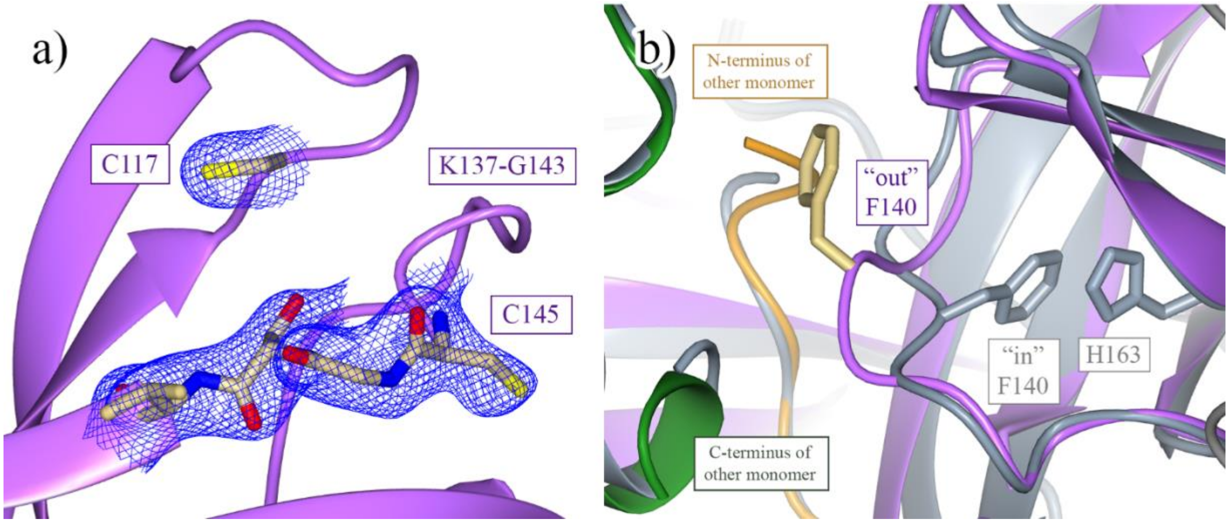

Supplementary Figure 8 – Reduced Structure of the H163A Mpro Mutant. (a) Soaking the H163A Mpro crystals with 20mM TCEP reduces the C117-C145 disulfide bond, which returns the active-site loop to a more WT-like state. The map was rendered at 1.2 σ $2F_o$-F_c. (b) Despite reduction of this disulfide bond, the enzyme remains in an inactive state due to the F140 side chain being in the outward position, with a similar conformation to the disulfide-bonded H163A structure depicted in Fig. 5a in the main text. The WT structure (PDB 7CAM) is shown in grey for comparison. Interestingly, the N-terminus of the reduced H163A structure is in a WT-like conformation whereas the disulfide-bonded H163A structure has its N-terminus rotated approximately 90° from the WT conformation (Fig. 4cd in the main text).

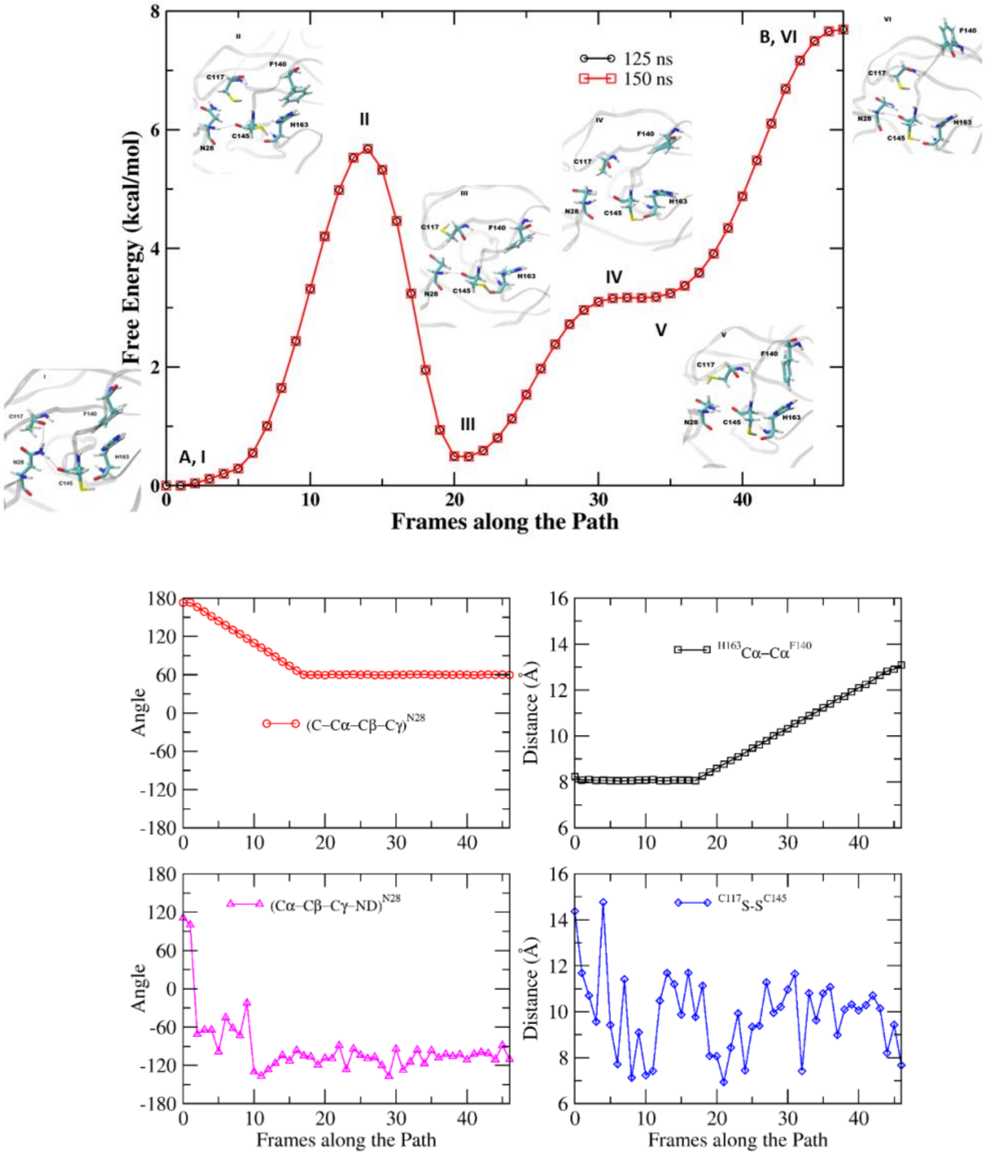

Supplementary Figure 9 – MFEP for WT model. (top half) (A, I) Conformation of WT structure; (II) transition state corresponding to the rotation of the N28 side chain; (III) minimum corresponding to the conformation after rotation of N28 dihedral; (IV) transition state corresponding to the dissociation of π-π stacking; (V) minimum corresponding to the C-H-π interaction between F140 and H163; (B,VI) Conformation corresponding to the mutated crystal structure. (bottom half) Change in dihedral angles of the N28 side chain happens first then Cα distance between H163 and F140 increases. The S-S distance between C117 and C145 goes from 14 to 8 Å along the MFEP.

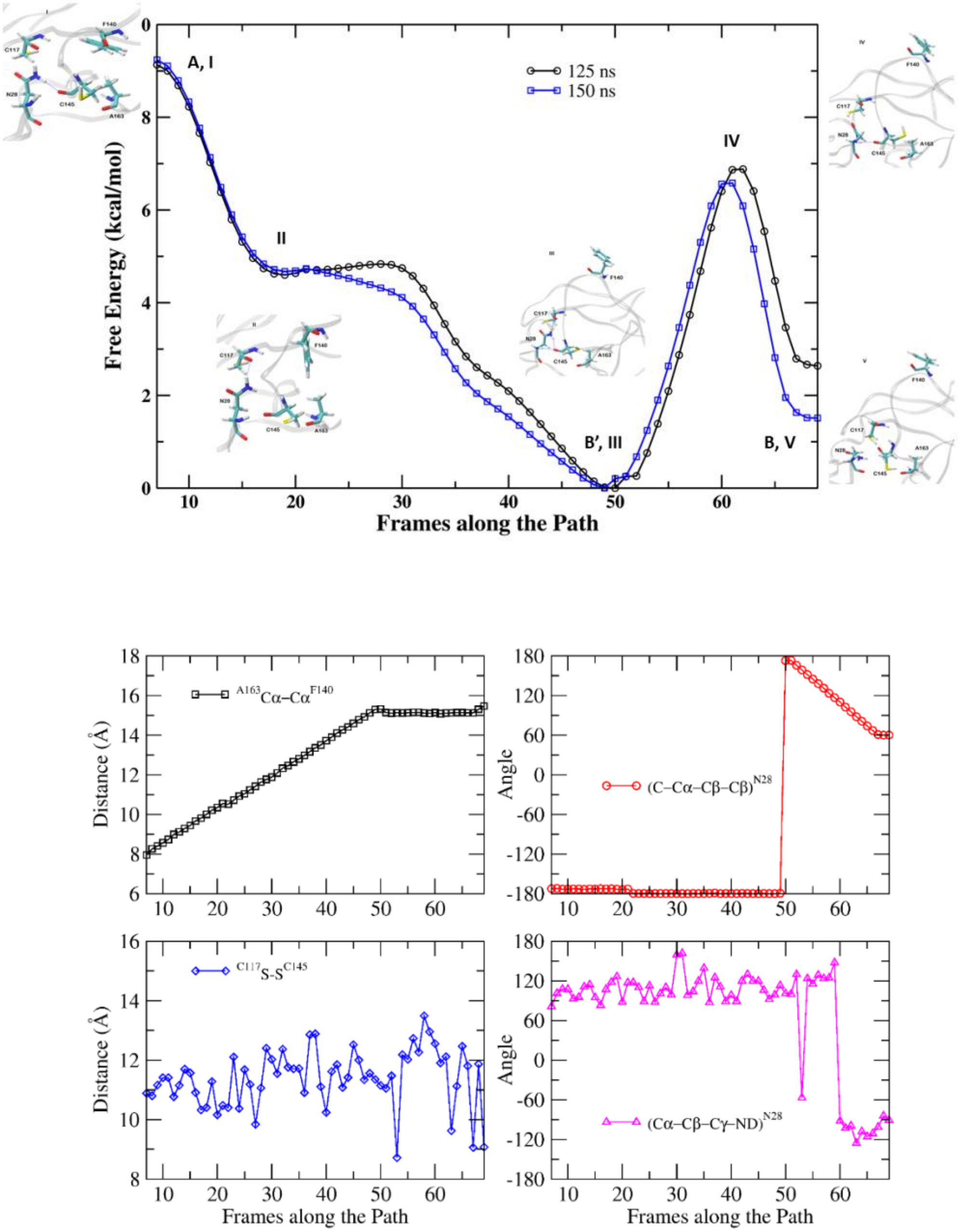

Supplementary Figure 10 – MFEP for H163A model. (top half) (A, I) Conformation corresponding to the WT structure for H163A mutated system; (II) Intermediated conformation along the dissociation of Cα distance between A163 and F140; (B', III) Conformation corresponding to elongated Cα distance between A163 and F140; (IV) Transition state conformation corresponding to the rotation of N28 side chain; (B, V) Conformation corresponding to the mutant crystal structure. (bottom half) Cα distance between H163 and F140 increases first followed by rotation of the N28 side chain. The S-S distance between C117 and C145 goes from 11 to 9 Å along the MFEP.

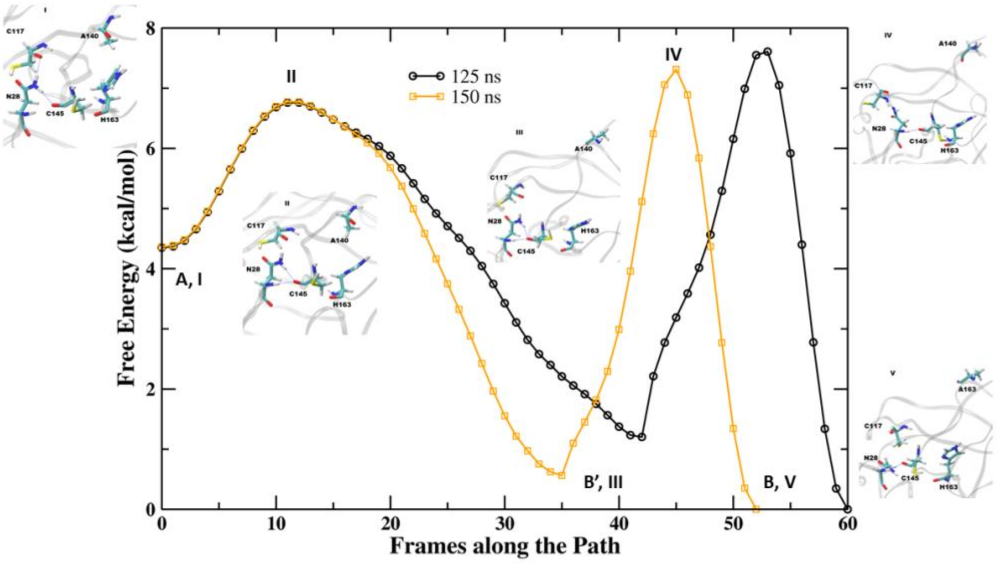

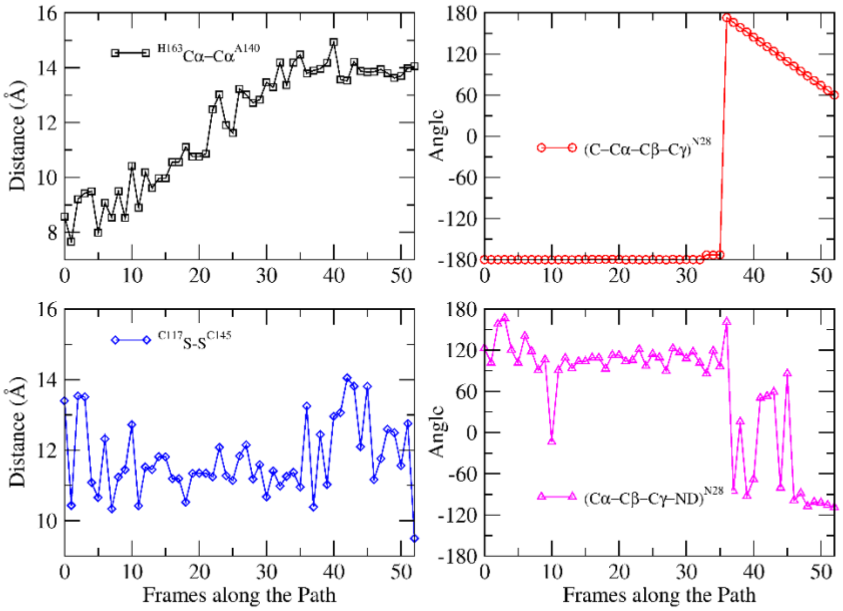

Supplementary Figure 11 – MFEP for F140A model. (top half) (A, I) Conformation corresponding to the WT structure for F140A system; (II) Transition state conformation along the dissociation of Cα distance between H163 and A140; (B', III) Conformation corresponding to elongated Cα distance between H163 and A140; (IV) Transition state conformation corresponding to the rotation of the N28 side chain; (B, V) Conformation corresponding to the mutant crystal structure. (bottom half) Cα distance between H163 and F140 increases first then the rotation of the N28 side chain occurs. The S-S distance between C117 and C145 goes from 13 to 9.5 Å along the MFEP.

.

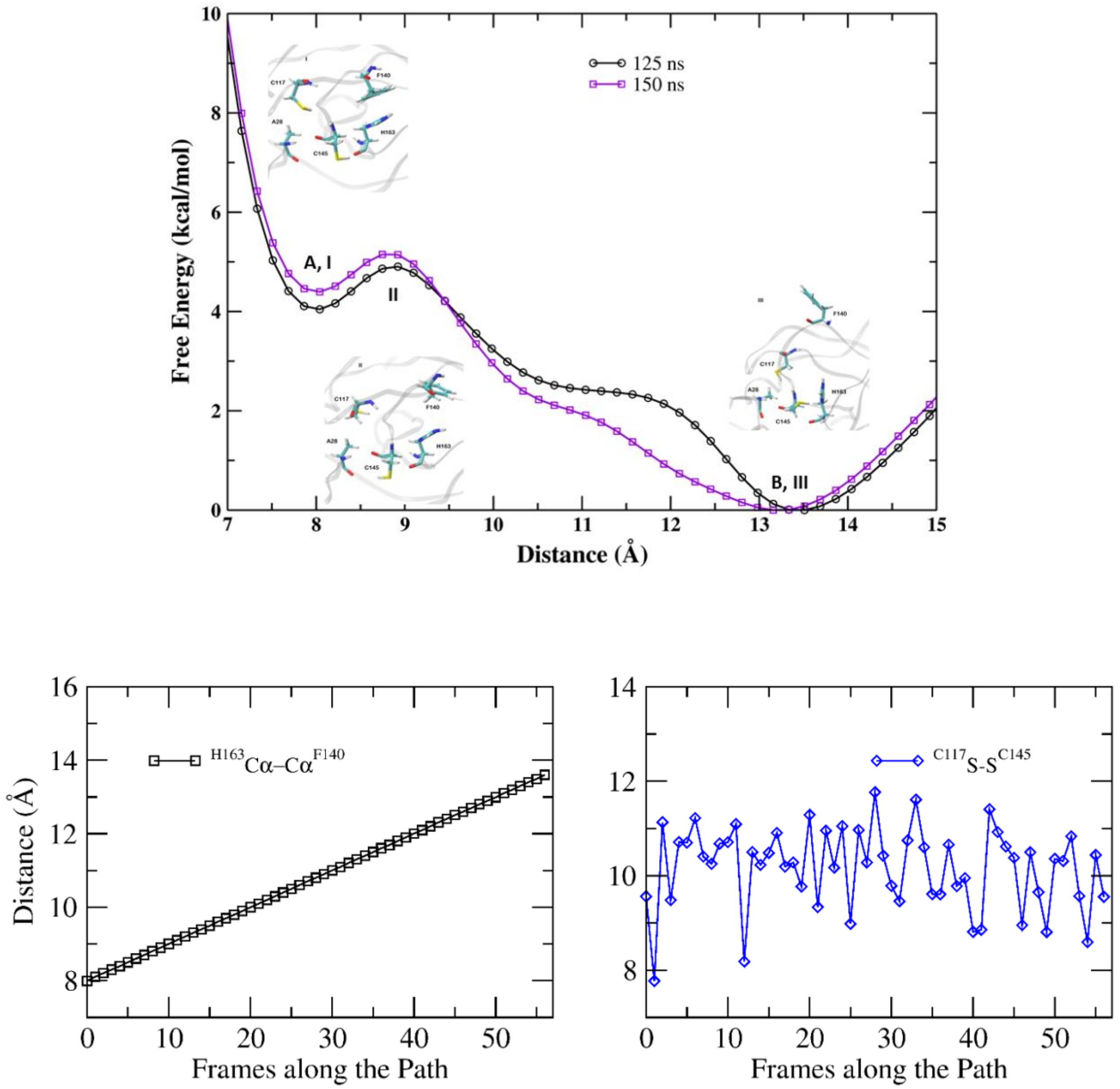

Supplementary Figure 12 – MFEP for N28A model. (top half) (A, I) Conformation corresponding to the WT structure for N28A system; (II) Transition state corresponding to the dissociation of π-π stacking and dissociation of Cα distance between H163 and F140; (B, III) Conformation corresponding to the mutant crystal structure. (bottom half) S-S distance between C117 and C145 fluctuates between 9 and 11 Å along the MFEP path.

Supplementary Table 1 – Data Collection and Refinement Statistics.

Structure description and PDB ID	H163A Mpro Cocrystallized with GC376 (**8DD6**)	H163A Mpro (**8DDL**)	H163A Mpro Soaked with 20mM TCEP (**8SG6**)
Wavelength (Å)	1.1271	0.9686	0.9686
Resolution range	54.97 – 2.30 (2.38 – 2.30)	56.54 – 1.94 (2.01 – 1.94)	57.37 – 2.49 (2.58 – 2.49)
Space group	I2	$P2_12_12_1$	$P2_12_12_1$
Unit cell (Å)	44.92 52.84 111.74 β = 100.3º	67.83 101.46 102.34	68.87 103.41 103.70
Total reflections	74279 (3750)	711101 (36076)	350104 (17765)
Unique reflections	11561 (558)	52678 (2569)	26455 (1313)
Multiplicity	6.4 (6.72)	13.50 (14.04)	13.23 (13.53)
Completeness (%)	99.76 (98.27)	99.48 (95.00)	99.35 (94.74)
Mean I/σ(I)	5.8 (0.5)	6.4 (0.5)	8.6 (0.5)
Wilson B-factor	36.06	28.34	56.39
R_{merge}	0.175 (0.895)	0.169 (1.082)	0.144 (1.909)
R_{meas}	0.191 (0.971)	0.176 (1.122)	0.150 (1.985)
R_{pim}	0.075 (0.374)	0.047 (0.297)	0.041 (0.539)
$CC_{1/2}$	0.991 (0.814)	0.996 (0.759)	0.998 (0.616)
Number of reflections used in refinement	11560 (1134)	52608 (4940)	26352 (2452)
Number of reflections used for R_{free}	579 (46)	2661 (241)	1339 (99)
R_{work}	0.1973 (0.2767)	0.1683 (0.2199)	0.1929 (0.3160)
R_{free}	0.2504 (0.3570)	0.2067 (0.2679)	0.2399 (0.3619)
Number of atoms	2499	5174	4638
Protein	2416	4729	4638
Ligands	4	69	14
Water	79	376	50
B-factors (Å^2)	43.51	35.35	77.32
Protein	43.57	34.76	77.52
Ligands	49.35	53.16	70.79
Water	41.34	40.90	59.53
Root mean square deviations			
Bonds (Å)	0.002	0.005	0.003
Angles (º)	0.49	0.76	0.66
Rotamer outliers (%)	0	0.56	0
Clashscore	1.48	5.05	6.42
Ramachandran (%)			
Favored	98.68	97.81	94.78
Allowed	0.99	2.19	5.05
Outliers	0.33	0	0.17

Supplementary Table 2 – Summary of Small-Angle X-ray Scattering Data.

Concentration (mg/mL)	0.25	0.5	1.0	3.0	6.3
General Information					
Instrument	CHESS BioSAXS ID7A				
Wavelength	1.1013 Å				
q-measurement range	0.00584 – 0.50081				
Exposure time	1 second				
Number of exposures	15				
Temperature	25 ºC				
Guinier analysis					
I(0) (Arb.)	0.00358	0.00844	0.02000	0.06000	0.1400
R_g (Å)	24.18	25.33	26.2	26.25	25.87
q-range (Å^{-1})	0.0185 – 0.0556	0.0148 – 0.0518	0.0138 – 0.0508	0.0133 – 0.0503	0.0133 – 0.0503
q_{min}*R_g	0.448	0.375	0.362	0.349	0.344
q_{max}*R_g	1.344	1.313	1.332	1.321	1.302
χ^2	0.696	0.914	0.981	0.997	0.996
P(r) analysis					
I(0) (Arb.)	0.00362	0.00849	0.02000	0.06000	0.1400
R_g (Å)	25.42	25.76	26.11	26.38	26.06
D_{max} (Å)	78.0	82.0	82.0	85.0	77.0
χ^2	0.901	0.857	0.886	0.966	0.863
q-range (Å^{-1})	0.0185 – 0.2199	0.0148 – 0.3007	0.0138 – 0.3502	0.0133 – 0.2500	0.0106 – 0.2301
Volume analysis					
V_p (kDa)	68.5	57.5	60.7	69.4	70.2
V_c (kDa)	48.8	49.6	51.7	57.7	58.5
SASBDB ID	SASDSP5	SASDSQ5	SASDSR5	SASDSS5	SASDST5

Supplementary Discussion – Summary of the Working Model for Generating the Oxidized, Disulfide-Bonded Mpro Conformation.

In summary, the specifics of the working model for the increased energetic favorability of the oxidized structural state are as follows:

- The H163A mutation results in the loss of the face-to-face π-stacking interaction between H163 and F140 (Fig. 5a)
- The energetic barrier between the inward and outward conformations of F140 and the S139-S147 loop is reduced due to destabilization of the inward conformation (Fig. 6)
- As the F140 side chain normally resides in a hydrophilic environment, upon adopting this outward position, residues S147, H172, Y118, and Y126 rearrange to form a new hydrogen bonding network alongside two new water molecules (Fig. 5c)
- Rearrangement of the active-site loop (S139-S147) allows for the active-site C145 to come in close proximity to C117 (compare Fig. 1b with Fig. 3a)
- A disulfide bond between C117 and C145 forms to stabilize this outward conformation and is structurally concomitant with the rotation of the N28 side chain (Fig. 3a)
- A NOS bridge between K61 and C22 is seen in one of the two protomers (Fig. 4ab)
- The N-terminus of one of the protomers is threaded 90° from its wild type orientation as the active-site loop, and in particular F140, sterically occludes the original position of the N-terminus (Fig. 4cd)
- The disulfide bond between C117-C145 is reversible under reducing conditions, as evident by kinetic data (Supplementary Fig. 2) and the mutant structure in complex with GC376 (Fig. 2)
- An energetically relaxed, wild-type-like conformation of the beta strand containing the active-site cysteine (S144-V148) is seen when the disulfide bond is broken (Fig. 3bc)
- The inward to outward conformation change rarely occurs the wild type enzyme as, in the presence of reducing agent (i.e. in normal physiological conditions), the C117-C145 disulfide bond is readily broken and the S139-S147 loop is restructured back into the inward conformation as F140 is easily stabilized by H163 (Fig. 6)
- *In silico* metadynamics simulations of the F140A and N28A mutants showed similar conformational behavior to the H163A mutant (Fig. 6), suggesting that these residues play an important role in stabilizing the WT conformation

Supplementary References

1. Laskowski, R. A. & Swindells, M. B. LigPlot+: Multiple ligand-protein interaction diagrams for drug discovery. *J Chem Inf Model* **51**, 2778–2786 (2011).

2. Lee, J. *et al.* Crystallographic structure of wild-type SARS-CoV-2 main protease acyl-enzyme intermediate with physiological C-terminal autoprocessing site. *Nat Commun* **11**, (2020).

3. Kikhney, A. G., Borges, C. R., Molodenskiy, D. S., Jeffries, C. M. & Svergun, D. I. SASBDB: Towards an automatically curated and validated repository for biological scattering data. *Protein Science* **29**, 66–75 (2020).

4. Hopkins, J. B., Gillilan, R. E. & Skou, S. BioXTAS RAW: improvements to a free open-source program for small-angle X-ray scattering data reduction and analysis. *J Appl Crystallogr* **50**, 1545–1553 (2017).

5. Svergun, D. I. Determination of the Regularization Parameter in Indirect-Transform Methods Using Perceptual Criteria. *J. Appl. Cryst* **25**, 495–503 (1992).

6. Schneidman-Duhovny, D., Hammel, M., Tainer, J. A. & Sali, A. Accurate SAXS Profile Computation and its Assessment by Contrast Variation Experiments. *Biophys J* **105**, 962–974 (2013).

7. McNicholas, S., Potterton, E., Wilson, K. S. & Noble, M. E. M. Presenting your structures: The CCP4mg molecular-graphics software. *Acta Crystallogr D Biol Crystallogr* **67**, 386–394 (2011).

8. Pettersen, E. F. *et al.* UCSF Chimera - A visualization system for exploratory research and analysis. *J Comput Chem* **25**, 1605–1612 (2004).
