# Supplementary material for: The H163A mutation unravels an oxidized conformation of the SARS-CoV-2 main protease
Source: Nat Commun. 2023 Sep 12;14:5625. doi: 10.1038/s41467-023-40023-4 (PMC10497556; doi:10.1038/s41467-023-40023-4)
Supplement: Supplementary file 3 — Description of Additional Supplementary Files [file 41467_2023_40023_MOESM3_ESM.docx]

**Description of Additional Supplementary Files**

File Name: Supplementary Data 1
Description: This file contains the initial and final coordinates of the WT Mpro system during classical MD simulation. Both the coordinates are provided in the same file as Models 1 (initial) and 2 (final).

File Name: Supplementary Data 2
Description: This file contains the initial and final coordinates of the H163A Mpro system during classical MD simulation. Both the coordinates are provided in the same file as Models 1 (initial) and 2 (final).

File Name: Supplementary Data 3
Description: This file contains the initial and final coordinates of the F140A Mpro system during classical MD simulation. Both the coordinates are provided in the same file as Models 1 (initial) and 2 (final).

File Name: Supplementary Data 4
Description: This file contains the initial and final coordinates of the N28A Mpro system during classical MD simulation. Both the coordinates are provided in the same file as Models 1 (initial) and 2 (final).

File Name: Supplementary Data 5
Description: This file contains the initial and final coordinates of the WT Mpro system from the metadynamics MD simulation. Both the coordinates are provided in the same file as Models 1 (initial) and 2 (final).

File Name: Supplementary Data 6
Description: This file contains the initial and final coordinates of the H163A Mpro system from the metadynamics MD simulation. Both the coordinates are provided in the same file as Models 1 (initial) and 2 (final).

File Name: Supplementary Data 7
Description: This file contains the initial and final coordinates of the F140A Mpro system from the metadynamics MD simulation. Both the coordinates are provided in the same file as Models 1 (initial) and 2 (final).

File Name: Supplementary Data 8
Description: This file contains the initial and final coordinates of the N28A Mpro system from the metadynamics MD simulation. Both the coordinates are provided in the same file as Models 1 (initial) and 2 (final).

File Name: Supplementary Data 9
Description: This file contains the coordinates for all the structures sampled along the free energy path of metadynamics simulation for the WT Mpro model provided in Supplementary Figure 9. The coordinates are provided for the points marked as I-VI in this figure. All the coordinates are aligned against point I.

File Name: Supplementary Data 10
Description: This file contains the coordinates for all the structures sampled along the free energy path of metadynamics simulation for the H163A Mpro model provided in Supplementary Figure 10. The coordinates are provided for the points marked as I-V in this figure. All the coordinates are aligned against point I.

File Name: Supplementary Data 11
Description: This file contains the coordinates for all the structures sampled along the free energy path of metadynamics simulation for the F140A Mpro model provided in Supplementary Figure 11. The coordinates are provided for the points marked as I-V in this figure. All the coordinates are aligned against point I.

File Name: Supplementary Data 12
Description: This file contains the coordinates for all the structures sampled along the free energy path of metadynamics simulation for the N28A Mpro model provided in Supplementary Figure 11. The coordinates are provided for the points marked as I-III in this figure. All the coordinates are aligned against point I.
