# Supplementary material for: The H163A mutation unravels an oxidized conformation of the SARS-CoV-2 main protease
Source: Nat Commun. 2023 Sep 12;14:5625. doi: 10.1038/s41467-023-40023-4 (PMC10497556; doi:10.1038/s41467-023-40023-4)
Supplement: Supplementary file 16 — Reporting Summary [file 41467_2023_40023_MOESM16_ESM.pdf]

# nature portfolio

Corresponding author(s): Aravindhan Ganesan
Todd Holyoak

Last updated by author(s): July 3, 2023

# Reporting Summary

Nature Portfolio wishes to improve the reproducibility of the work that we publish. This form provides structure for consistency and transparency in reporting. For further information on Nature Portfolio policies, see our Editorial Policies and the Editorial Policy Checklist.

## Statistics

For all statistical analyses, confirm that the following items are present in the figure legend, table legend, main text, or Methods section.

| n/a | Confirmed | |
|---|---|---|
| ☐ | ☒ | The exact sample size (*n*) for each experimental group/condition, given as a discrete number and unit of measurement |
| ☐ | ☒ | A statement on whether measurements were taken from distinct samples or whether the same sample was measured repeatedly |
| ☒ | ☐ | The statistical test(s) used AND whether they are one- or two-sided<br>*Only common tests should be described solely by name; describe more complex techniques in the Methods section.* |
| ☒ | ☐ | A description of all covariates tested |
| ☒ | ☐ | A description of any assumptions or corrections, such as tests of normality and adjustment for multiple comparisons |
| ☐ | ☒ | A full description of the statistical parameters including central tendency (e.g. means) or other basic estimates (e.g. regression coefficient) AND variation (e.g. standard deviation) or associated estimates of uncertainty (e.g. confidence intervals) |
| ☒ | ☐ | For null hypothesis testing, the test statistic (e.g. *F*, *t*, *r*) with confidence intervals, effect sizes, degrees of freedom and *P* value noted<br>*Give P values as exact values whenever suitable.* |
| ☒ | ☐ | For Bayesian analysis, information on the choice of priors and Markov chain Monte Carlo settings |
| ☒ | ☐ | For hierarchical and complex designs, identification of the appropriate level for tests and full reporting of outcomes |
| ☒ | ☐ | Estimates of effect sizes (e.g. Cohen's *d*, Pearson's *r*), indicating how they were calculated |

*Our web collection on statistics for biologists contains articles on many of the points above.*

## Software and code

Policy information about availability of computer code

Data collection

The following software were used for data collection:
GROMACS-2019.6
PLUMED-2.6.2.

Further details of data collection are provided below:

All MD simulations were carried out using the GROMACS-2019.6 program and AMBERff14SB forcefield for describing the model systems. The well-tempered metadynamics simulations were carried out using the PLUMED-2.6.2 patch available for the GROMACS-2019.6.

Diffraction data were collected at the Cornell High-Energy Synchrotron Source (CHESS) ID7B2 beamline on a Detectris Pilatus3 S 6M. High-throughput crystallization trials for the H163A mutant were carried out with commercially available screens in small-volume sitting-drop trays using a Crystal Gryphon LCP robot (Art Robbins Instruments; Sunnyvale, CA, USA).

SAXS data were collected at the Cornell High-Energy Synchrotron Source (CHESS) ID7A beamline using an X-ray wavelength of 1.1013 Å and an EIGER 4M detector.

The Tecan Infinite M1000 plate reader (360 nm excitation, 490 nm emission, 5 nm bandwidths) was used for fluorescent kinetic assay.

A MicroCal VP-DSC MicroCalorimeter (Northampton, MA, USA) was used for collecting DSC data.

Data analysis

The following software were used for data analysis:
VMD-1.9.3 software
MEPSA-v1.4 software
Grace-5.1.25
DIALS (version 3.8.0)
CCP4i suite (version 8.0.009)
AIMLESS (version 0.7.9).
MOLREP version 11.9.02
phenix.refine (version 1.20.1_4487)
COOT (version 0.8.9.2).
MolProbity (version 4.5.2).
RAW (version 2.1.4).
GNOM (ATSAS package, version 3.2.1),
FoXS (web server, accessed April 17th, 2023).
LigPlot+ (version 2.2).

Further details on the application of these programs in data analysis are provided below:
The minimum free-energy paths corresponding to state transitions were computed using the MEPSA-v1.4 software. All structure visualization and analyses of simulation trajectories were performed using VMD-1.9.3 software and the plots were generated using Grace-5.1.25 plotting tool and MEPSA-v1.4 software.

Data were indexed, integrated, and scaled with DIALS50 (version 3.8.0) and imported into CCP4i suite (version 8.0.009) with AIMLESS (version 0.7.99). Molecular replacement (MOLREP version 11.9.02) for all structures were done with a high-resolution WT Mpro model (PDB 7ALH). Refinement was done using phenix.refine (Version 1.20.1_4487) in conjunction with manual model building in COOT (0.8.9.2). Translation-libration-screw parameters were automatically determined and used by phenix.refine (Version 1.20.1_4487) for all structures. Model geometry was analysed and optimized based on suggestions by MolProbity (Version 4.5.2).

SAXS images were azimuthally averaged and buffer subtracted in RAW (version 2.1.4). Processed data were subsequently analysed in RAW (version 2.1.4), GNOM (ATSAS package, version 3.2.1), and FoXS (web server, accessed April 17th, 2023). 2D interaction map for ligand-bound enzyme complexes (WT and H163A) were generated using LipPlot (version 2.2).

For manuscripts utilizing custom algorithms or software that are central to the research but not yet described in published literature, software must be made available to editors and reviewers. We strongly encourage code deposition in a community repository (e.g. GitHub). See the Nature Portfolio guidelines for submitting code & software for further information.

# Data

Policy information about availability of data

All manuscripts must include a data availability statement. This statement should provide the following information, where applicable:

- Accession codes, unique identifiers, or web links for publicly available datasets
- A description of any restrictions on data availability
- For clinical datasets or third party data, please ensure that the statement adheres to our policy

Data availability

Raw experimental data and metadynamics trajectory files that are needed to recapitulate the results of this paper can be requested from the authors. Source data are provided with this paper. The WT Mpro structure used for all the modelling and simulation in this work are available with the PDB accession code 7JUN [https://doi.org/10.2210/pdb7JUN/pdb]. The initial and final coordinates of the WT and mutant models from classical MD simulations in this work are provided in the Supplementary Data 1-4. The initial and final coordinates of the WT and mutant models from metadynamics simulations in this work are provided in the Supplementary Data 5-8. The conformations sampled along the free-energy paths of the WT and mutant Mpro models described in Supplementary Figures 9-12 are provided as single aligned PDB files for each system in the Supplementary Data 9-12. The previously published crystal structure of WT SARS-CoV-2 Mpro used in this work for molecular replacement is available with the PDB accession code 7ALH [https://doi.org/10.2210/pdb7ALH/pdb]. Maps and models for a variety of Mpro structures that were referenced in the text and figures but were not directly used to process data can be found in the PDB with the following accession codes: 6XB0 [https://doi.org/10.2210/pdb6XB0/pdb], 6XMK [https://doi.org/10.2210/pdb6XMK/pdb], 7JR4 [https://doi.org/10.2210/pdb7JR4/pdb], 3FZD [https://doi.org/10.2210/pdb3FZD/pdb], 7TGR [https://doi.org/10.2210/pdb7TGR/pdb], and 7BB2 [https://doi.org/10.2210/pdb7BB2/pdb]. The maps and models for the GC376-bound H163A Mpro, apo H163A Mpro, and reduced H163A Mpro structures are available in the PDB accession codes, 8DD6 [http://doi.org/10.2210/pdb8DD6/pdb], 8DDL [https://doi.org/10.2210/pdb8ddl/pdb], and 8SG6 [https://doi.org/10.2210/pdb8SG6/pdb], respectively. SAXS data for the 0.25, 0.5, 1.0, 3.0, and 6.3 mg/mL H163A Mpro have been deposited in the SASBDB with database IDs of SASDSP5 [https://www.sasbdb.org/data/SASDSP5], SASDSQ5 [https://www.sasbdb.org/data/SASDSQ5], SASDSR5 [https://www.sasbdb.org/data/SASDSR5], SASDSS5 [https://www.sasbdb.org/data/SASDSS5], and SASDST5 [https://www.sasbdb.org/data/SASDST5], respectively.

# Research involving human participants, their data, or biological material

Policy information about studies with human participants or human data. See also policy information about sex, gender (identity/presentation), and sexual orientation and race, ethnicity and racism.

| | |
|---|---|
| Reporting on sex and gender | N/A |
| Reporting on race, ethnicity, or | N/A |

| | |
|---|---|
| other socially relevant groupings | |
| Population characteristics | N/A |
| Recruitment | N/A |
| Ethics oversight | N/A |

Note that full information on the approval of the study protocol must also be provided in the manuscript.

# Field-specific reporting

Please select the one below that is the best fit for your research. If you are not sure, read the appropriate sections before making your selection.

- [x] Life sciences
- [ ] Behavioural & social sciences
- [ ] Ecological, evolutionary & environmental sciences

For a reference copy of the document with all sections, see nature.com/documents/nr-reporting-summary-flat.pdf

# Life sciences study design

All studies must disclose on these points even when the disclosure is negative.

| | |
|---|---|
| Sample size | Not applicable. No sample size calculation was done. The replicates at each substrate concentration for the enzyme (n=3) provide statistical meaningful mean+- SD values. |
| Data exclusions | There is no data exclusion in this study. |
| Replication | The activity assays for the Mpro were performed in triplicates. The activity assay of Mpro has been attempted more than 3 times with all attempts being successful. |
| Randomization | Experiments are concerned with the characterization of recombinant Mpro protein. Randomization is thus,not applicable for these basic in vitro biophysical experiments. |
| Blinding | Experiments are concerned with the characterization of recombinant Mpro protein. Blinding is thus,not applicable for these basic in vitro biophysical experiments. |

# Reporting for specific materials, systems and methods

We require information from authors about some types of materials, experimental systems and methods used in many studies. Here, indicate whether each material, system or method listed is relevant to your study. If you are not sure if a list item applies to your research, read the appropriate section before selecting a response.

## Materials & experimental systems

| n/a | Involved in the study | |
|---|---|---|
| x | | Antibodies |
| x | | Eukaryotic cell lines |
| x | | Palaeontology and archaeology |
| x | | Animals and other organisms |
| x | | Clinical data |
| x | | Dual use research of concern |
| x | | Plants |

## Methods

| n/a | Involved in the study | |
|---|---|---|
| x | | ChIP-seq |
| x | | Flow cytometry |
| x | | MRI-based neuroimaging |
